# Long-Term Antifungal Treatment in a Patient With Confirmed Allergic Bronchopulmonary Aspergillosis

**DOI:** 10.7759/cureus.72142

**Published:** 2024-10-22

**Authors:** Hassan Imran Aziz, Imran Aziz

**Affiliations:** 1 Internal Medicine, Wrightington, Wigan and Leigh Teaching Hospitals NHS Foundation Trust, Wigan, GBR; 2 Respiratory Medicine, Wrightington, Wigan and Leigh Teaching Hospitals NHS Foundation Trust, Wigan, GBR

**Keywords:** allergic bronchopulmonary aspergillosis (abpa), aspergillus fumigatus, asthma, respiratory disease, respiratory fungus

## Abstract

An allergic reaction to infections with Aspergillus fumigatus causes allergic bronchopulmonary aspergillosis (ABPA). This response often can worsen underlying symptoms of previously well-controlled diseases, such as asthma or cystic fibrosis. Due to the nature of the symptoms, patients with ABPA are often regarded as just having worsening asthma control or are treated for a different disease entirely. Even when diagnosed, the treatment is often formulaic and not tailored to the patient directly. This report will discuss the case of a 63-year-old man with a background of asthma who presented with recurrent chest infections, in which treatment with antibiotics and steroids did not resolve his symptoms. A diagnosis of ABPA was made following investigations, which has led to better control of his symptoms on long-term antifungal treatment.

## Introduction

Aspergillus fumigatus is known to cause an allergic reaction in patients with underlying respiratory illnesses, which can lead to allergic bronchopulmonary aspergillosis (ABPA) [1]. ABPA is most commonly associated with patients who have chronic underlying respiratory diseases, such as allergic asthma and cystic fibrosis. In such patients, the means of diagnosis is based on clinical history and investigations [2].

It is difficult to ascertain the presence of ABPA based on clinical symptoms alone due to its similarity to other underlying diseases, hence the need for further investigations, most significantly blood tests. These will most frequently show elevated findings of serum IgE, along with elevated serum levels specific to Aspergillus of both IgE and IgG [2]. Standard practice in treating ABPA involves a course of steroids and antifungal treatment, which is usually weaned down to a point where it is stopped within the year [2].

Here, we report the case of a patient who was repeatedly diagnosed with exacerbations of asthma before he was diagnosed with ABPA by the respiratory team based on his clinical history and investigations.

## Case presentation

A 63-year-old non-smoker, who was a retired school principal with a history of asthma, presented with recurrent episodes of cough and phlegm production. These were diagnosed as infective exacerbations of asthma and were treated with antibiotics and steroids, which only provided temporary symptomatic relief. On one occasion, he had an episode of hemoptysis; however, as this was resolved with the usual antibiotic and steroid therapy, no further investigations were deemed necessary at the time. Clinical examination revealed left-sided basal chest crackles on auscultation, but no wheeze was present.

Following nine months of recurrent chest infections, his GP and the acute medical team referred him to the respiratory team at the same time. The respiratory team arranged a chest X-ray, a high-resolution CT of the thorax, and further blood tests, including allergy testing.

**Figure 1:**
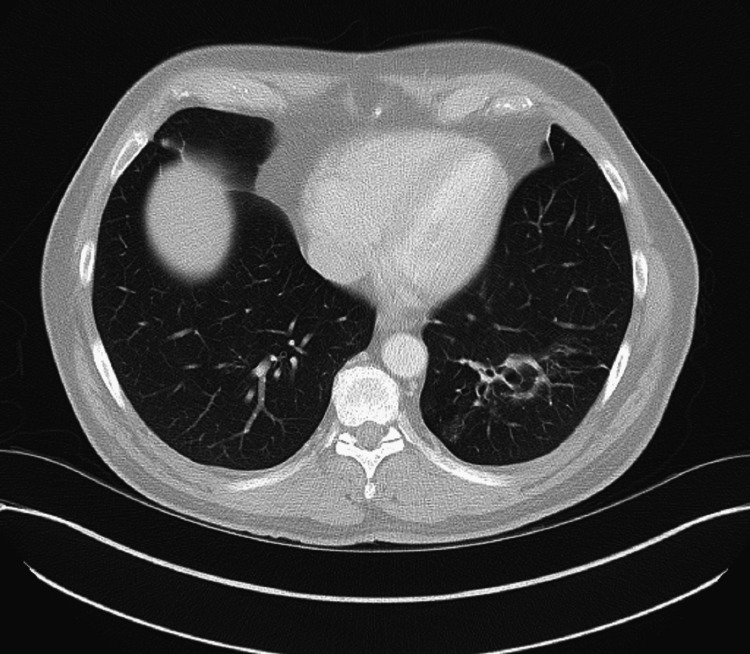
Our patient’s thoracic CT which showed shadowing and bronchiectasis in the left lower lung. Signed consent was obtained from the patient prior to using the image in the case report.

His blood tests showed he was allergic to dogs as he had a raised dog dander IgE of 49.60kU/L (normal values are less than 0.10kU/L), as well as mild eosinophilia of 0.6x10^9^/L (normal values are less than 0.4x10^9^/L). More concerningly, however, was that his total serum IgE result came back at over 6000kU/L (normal values are less than 114kU/L), his aspergillus IgG levels came back at over 200mgA/L (normal values are less than 40mgA/L), which was the highest result provided by the lab, and his Aspergillus IgE was 10.3kUA/L (normal values are less than 0.35kUA/L). His CT showed some bronchial wall thickening and bronchiectasis, mainly affecting the left lung (Figure 1).

Based on his investigation results, he was started on itraconazole 400mg daily, along with a course of steroids, which was weaned down to zero over a fortnight, and his chronic asthma treatment was continued.

Following treatment, his clinical symptoms and blood results showed improvement, and therefore his itraconazole was reduced to 300mg daily. With continued improvement in his symptoms, the dose decreased to 200mg daily. At that time, the aim was to stop itraconazole after 6-12 months of therapy. Unfortunately, his symptoms and blood results both worsened, and his dose of itraconazole was increased back to 400mg a day.

**Figure 2:**
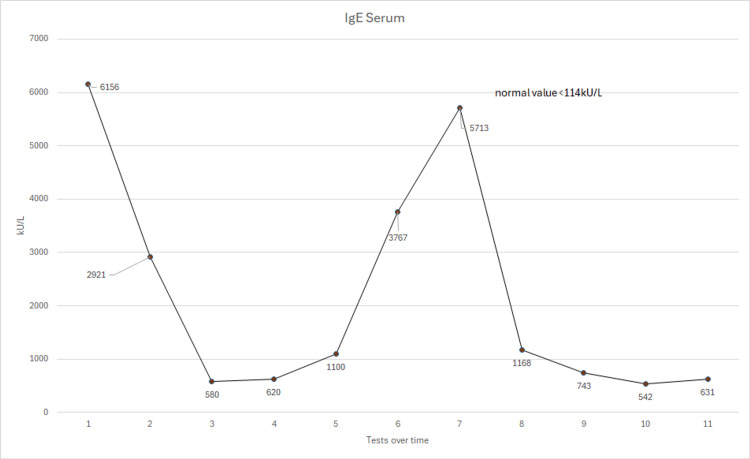
A graph showing his total serum IgE levels from 2013-2024

**Figure 3:**
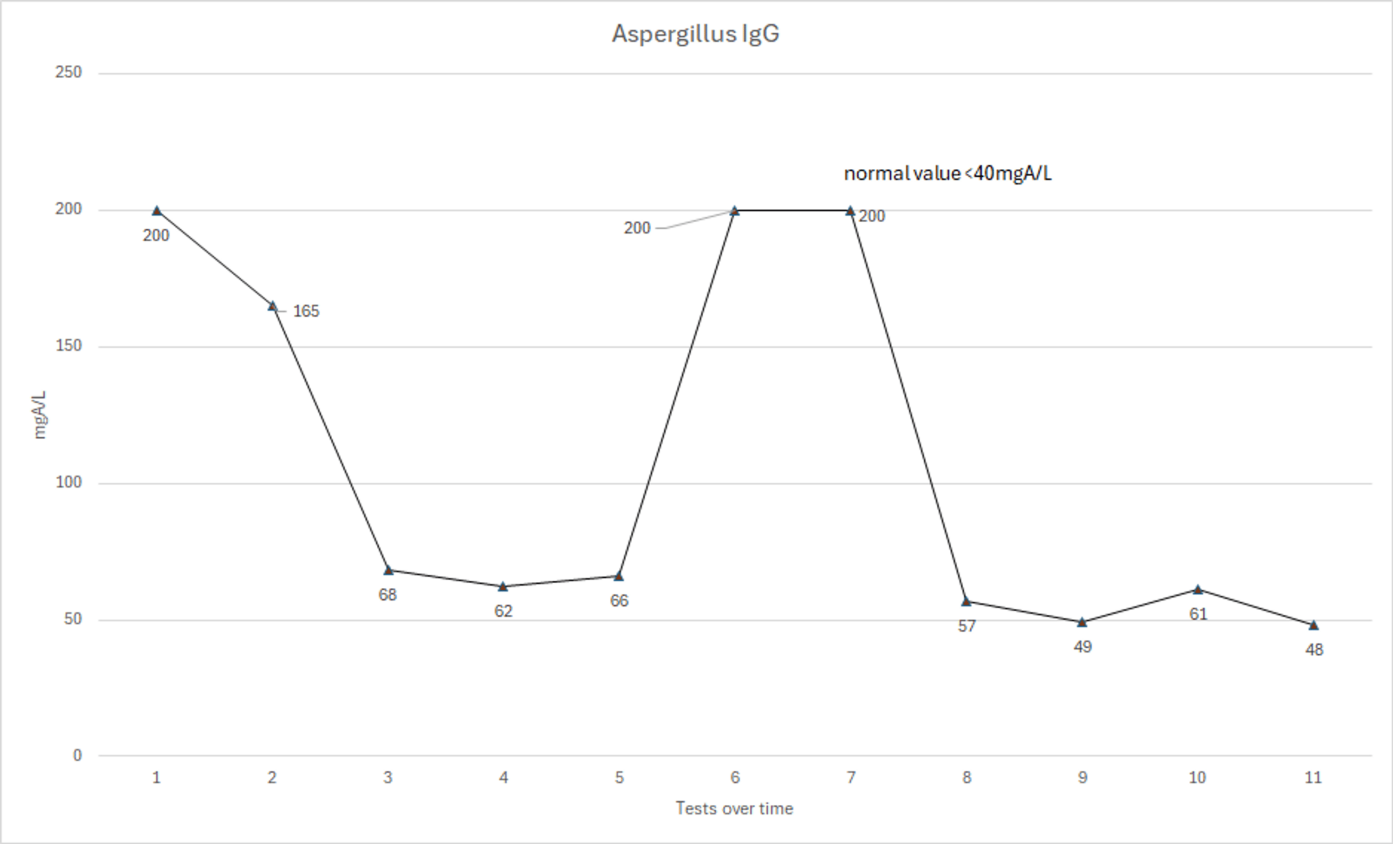
A graph showing his Aspergillus IgG levels from 2013-2024

**Figure 4:**
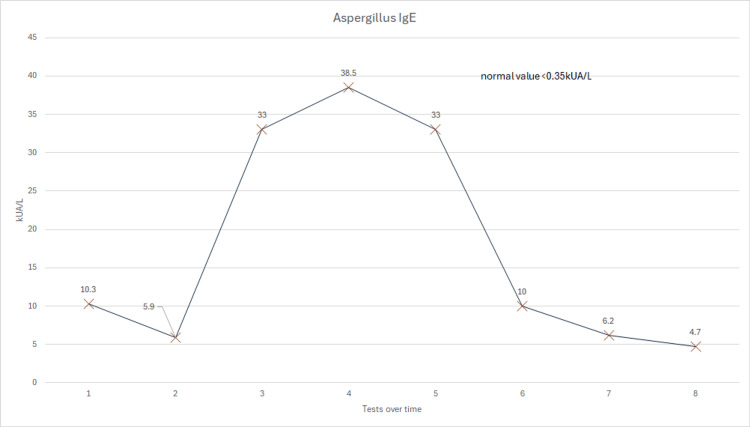
A graph showing his Aspergillus IgE levels from 2013-2024

However, despite his serum itraconazole level being within the therapeutic range, his blood showed worsening IgE and aspergillus serology, so the decision was made to increase his itraconazole dose to 600mg daily. Following marked improvement in his blood results and symptoms, his itraconazole dose has slowly been weaned to 400mg daily over the course of a year. Although the patient initially did not want to take high-dose therapy, it was noted that every time his itraconazole dose was decreased to below 400mg daily, his symptoms and serology worsened. Therefore, he has stayed on 400mg a day for almost a decade with very good control of his symptoms. He is seen yearly by a respiratory consultant, and his immunoglobulins, Aspergillus, and itraconazole levels are checked regularly, along with his renal and liver function tests, which have remained normal throughout, to ensure that his treatment remains optimized for him. Figures 2-4 show the patient's serological results over time.

## Discussion

In patients with asthma who suffer from recurrent chest infections, searching for an underlying cause is very important. Although most patients will have a viral or bacterial infection, if the patient is not responding to the usual therapy or only has a transient improvement, then one possible cause for these could be reactions to infections with or an allergy to Aspergillus. These infections can cause non-specific symptoms, such as a cough, phlegm production, shortness of breath, or chest pain, and auscultation can reveal wheezing, leading to other diagnoses being considered ahead of ABPA [2].

**Figure 5:**
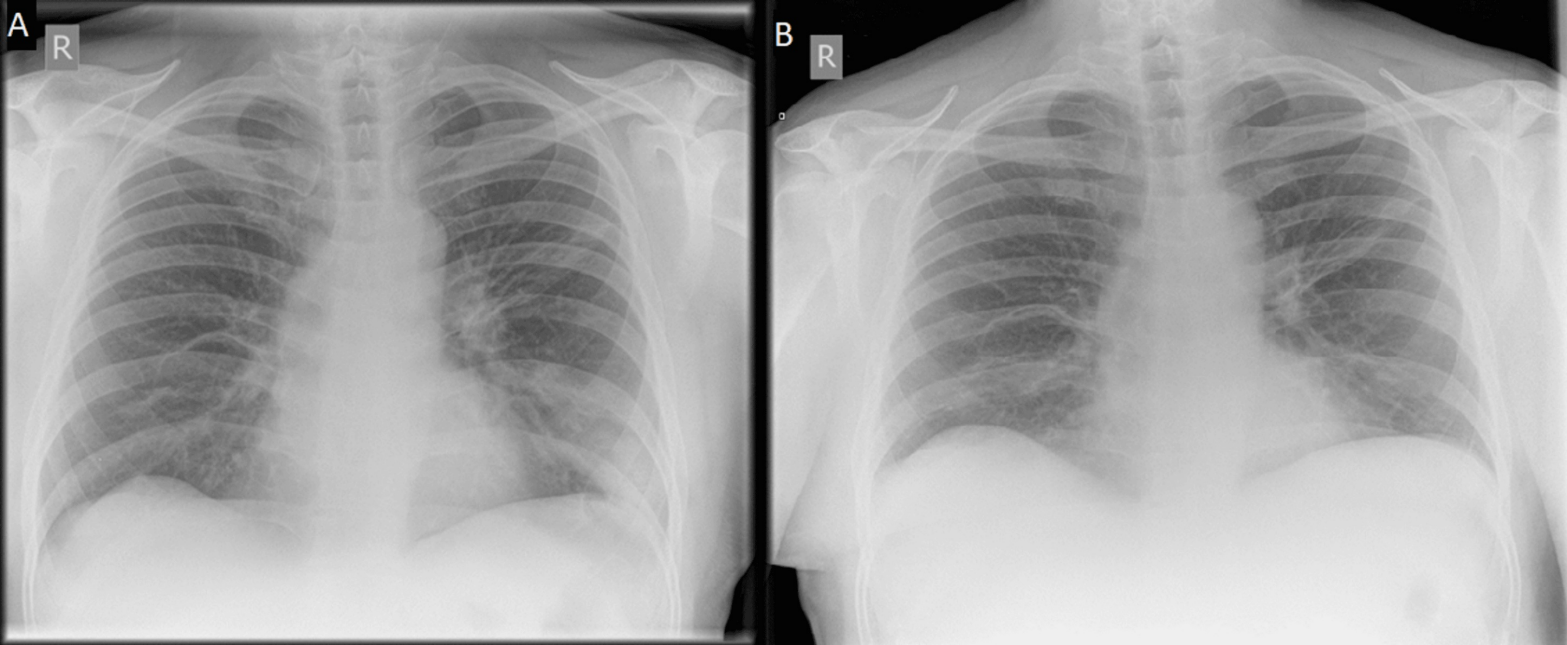
(A) PA chest x-ray of our patient showing shadowing in the left lower lobe. (B) Repeat PA chest x-ray showing the resolution of the shadowing in the left lower lobe following treatment. Signed consent was obtained from the patient prior to using the images in the case report.

Due to the non-specificity of symptoms, the diagnosis of ABPA can be suspected based on a combination of the clinical picture and findings from radiological and serological investigations. Often, it is noted that high levels of IgE and IgG specific to Aspergillus are found in the blood. Chest X-rays are not diagnostic in every scenario, but they most commonly demonstrate changes in the upper lobes; interestingly, in this particular patient, the changes in his chest X-ray were in his left lower zone, which then resolved with treatment (Figure 5A, 5B) [2].

The diagnosis of ABPA is commonly confirmed using the Modified Rosenberg-Patterson criteria. This states that if all seven criteria are met, then a diagnosis of ABPA can be confirmed, and if six are present, then a probable diagnosis of ABPA can be made [3]. All seven of these criteria were present in our patient (Table 1).

**Table 1:** Modified Rosenberg-Patterson criteria for the diagnosis of ABPA and their presence in our patient

Criteria	Present in this case
History of asthma	+
Eosinophilia	+
Raised specific IgE	+
Raised specific IgG	+
Raised serum IgE	+
History of pulmonary infiltrates	+
Bronchiectasis	+

Standard practice for the treatment of ABPA involves corticosteroids, usually prednisolone, and antifungal therapy, usually itraconazole, for 4-6 months, which is then decreased steadily over the next 4-6 months [4]. Antifungal treatment has been shown to reduce the required corticosteroid dose. It is especially useful in patients on high doses of corticosteroids or with a high frequency of exacerbations related to their underlying respiratory disease. Leaving patients on a low dose of steroids is sometimes considered as a way of preventing the disease from progressing [5].

In the case of our patient, the plan was for itraconazole to be reduced. However, this led to his symptoms worsening, as well as his blood results showing increased markers of disease. Therefore, his dose was increased despite initial attempts to taper, and he has been left on a long-term maintenance dose. Whilst this is not the conventional way of treating ABPA, this individualized approach has led to a decrease in exacerbations and related symptoms and has contributed towards improvements in his blood results. Most reassuringly, he has not required further treatment with prednisolone, which has a lot more long-term side effects than itraconazole [6,7].

Itraconazole is an antifungal agent that can lead to several side effects, including, but not limited to, hypersensitivity, and gastrointestinal symptoms such as diarrhea, heart failure, and vision disorders [7]. It can also cause QT interval prolongation; therefore, it is important to check the patient's ECG prior to starting therapy and ensure it is not co-administered with other drugs that can cause QT interval prolongation. Patients should be cautioned regarding this prior to starting treatment [8].

## Conclusions

This case shows how treatment for patients should not be based on a one-size-fits-all approach; rather, it should be tailored specifically to their own preferences, symptoms, and results. The common consensus in treating ABPA is that it is a finite course of itraconazole, but this case shows that long-term itraconazole may be a useful way to help improve symptoms and, therefore, quality of life. Further research should be undertaken to assess whether long-term low-dose antifungal therapy could be used alone rather than in combination with corticosteroids and antifungal therapy. We also recommend that further research into the long-term safety of antifungals be carried out.

In summary, treatment of ABPA is based on symptoms, results, and the expertise of the treating clinician, along with the wishes of the patients, who should be aware of the potential side effects of any treatment.
